# Image‐based investigation of lip aging features in a large number of Korean women

**DOI:** 10.1111/srt.13563

**Published:** 2024-01-09

**Authors:** Jung Yeon Seo, Sangseob Leem, Hanji Kim, Seung Won You, Yunkwan Kim, Nae Gyu Kang

**Affiliations:** ^1^ Research and Innovation Center, R&D Center LG H&H Seoul Republic of Korea

**Keywords:** lip aging, lip color, lip image, lip morphology, lip wrinkle

## Abstract

**Background:**

The lips play a significant role in shaping facial aesthetics. Due to the distinct attributes of lips in contrast to other facial skin, a unique approach is imperative for managing lip aging. We analyzed lip characteristics (morphology, wrinkles, and color) to investigate visual changes and distinctive attributes of aging lips.

**Methods:**

By utilizing image data processing methods, including facial landmark detection, pattern recognition, and color quantification, we extracted 11 lip characteristic indices (four morphological indices, four wrinkle indices, and three color indices) from high‐resolution images of 1000 Korean women aged 20–69. Correlation tests were conducted to assess the relationship between lip characteristic indices and age, and also between lip morphological and wrinkle indices.

**Results:**

Lip height significantly decreased, while lip width and lip ratio (lip width divided by the sum of the upper and lower lip height) significantly increased with aging. Lip wrinkles significantly increased with aging, whereas lip colors (redness and yellowness) decreased. The lip wrinkle indices, which are segmented for the first time in this study, exhibited significant correlations with lip width, and three of them additionally were correlated with lip ratio (*p* < 0.05). The results imply such morphological changes can be associated with wrinkle formation of human lips.

**Conclusion:**

The indices suggested in this study can be used for assessing lip aging characteristics, and the study results can contribute to deeper understanding of lip aging.

**Abbreviations**
LH: (lower horizontal)
LV: (lower vertical)
UH: (upper horizontal)
UV: (upper vertical)

## 1 INTRODUCTION

The lip is a prominent feature of the face and significantly contribute to an individual's facial aesthetics.^1^ The characteristics of lip skin differ from those of surrounding facial skin. For instance, the vermilion of the lips is comprised of a modified mucous membrane composed of non‐keratinized stratified squamous epithelium that is hairless and highly vascularized. Moreover, the vermilion lacks the typical skin appendages including hair follicles, sweat glands, and sebaceous glands.^2^, ^3^ Therefore, since the lips have different characteristics from the facial skin, a different angle of approach is required for lip aging care, and for this purpose, it is necessary to understand those changes underlying aging.

Several studies have investigated age‐related changes in lip. It has been known that with aging, the height of the upper lip decreases while the lip width increases.^4^, ^5^, ^6^ Additionally, studies have reported an increase in perioral region wrinkles with aging.^7^, ^8^, ^9^ However, most of lip aging studies were conducted on a small scale owing to relying on instrumental measurement methods.^7^, ^10^, ^11^ Additionally, there are many studies on perioral wrinkles (around the lips),^7^, ^12^ but as far as we know, there are few studies on wrinkles according to aging in the vermillion zone.^13^

Recently, due to the development of image data processing technology^14^, ^15^ and the increase in the ease of acquisition of high‐resolution image data, there has been a significant increase in reported research utilizing images. For instance, a method has been reported for extracting lip color using hyperspectral imaging technology.^16^ Furthermore, research with large‐scale image data has been actively emerging. In the most recent study, a considerable number of high‐resolution facial selfie images have been collected for the comparison of facial aging patterns between European and Chinese women.^17^ Similar to other research domains, in the field of dermatology, several studies have employed methods such as pattern recognition and deep learning to extract skin characteristics from images. For instance, two independent previous studies have applied methods such as hybrid Hessian filter^18^ and U‐net^19^ to extract wrinkles from images. However, there has been a lack of studies that focus on lips rather than other facial regions and conduct investigations into the patterns of aging through the analysis of extensive lip image data.

Our research aims to comprehensively understand the visual changes of lip aging using extensive image data. Therefore, we applied three kinds of image analysis techniques such as facial landmark detection, pattern recognition, and color quantification to lip images of 1000 Korean women aged between 20 and 69. With the 11 lip characteristics determined in this study, lip aging features such as morphology, wrinkles, and colors were investigated, and the correlation between the features was also analyzed.

## 2 MATERIALS AND METHODS

### 2.1 Study participants and facial image acquisition

**FIGURE 1:**
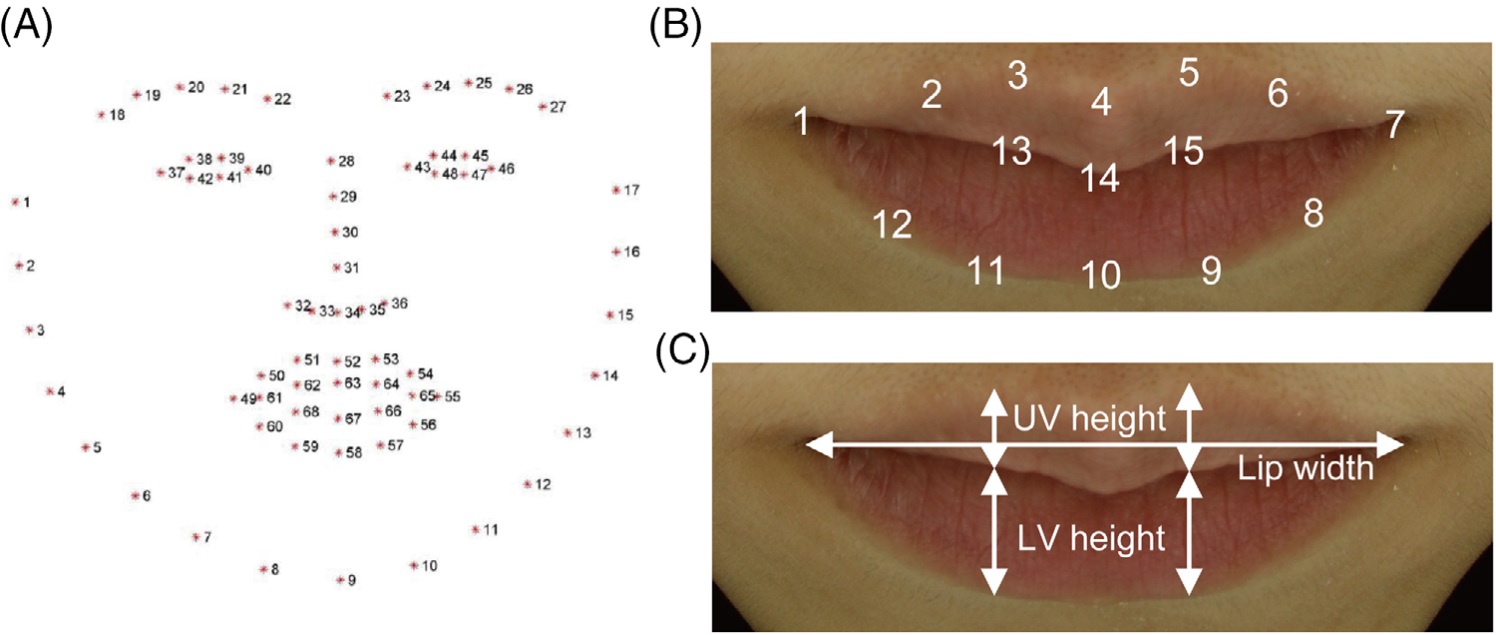
Details of the facial landmark detection and lip morphological indices. (A) An example image of facial landmark extraction taken from Sagonas et al.^39^ A total of 68 facial landmarks were extracted through this method. (B) Of the entire facial image, only the lip area was cropped, and the landmarks of the lip area were renumbered for convenience of explanation. (C) The three lip morphological indices, upper vertical (UV) height, lower vertical (LV) height, and lip width.

**FIGURE 2:**
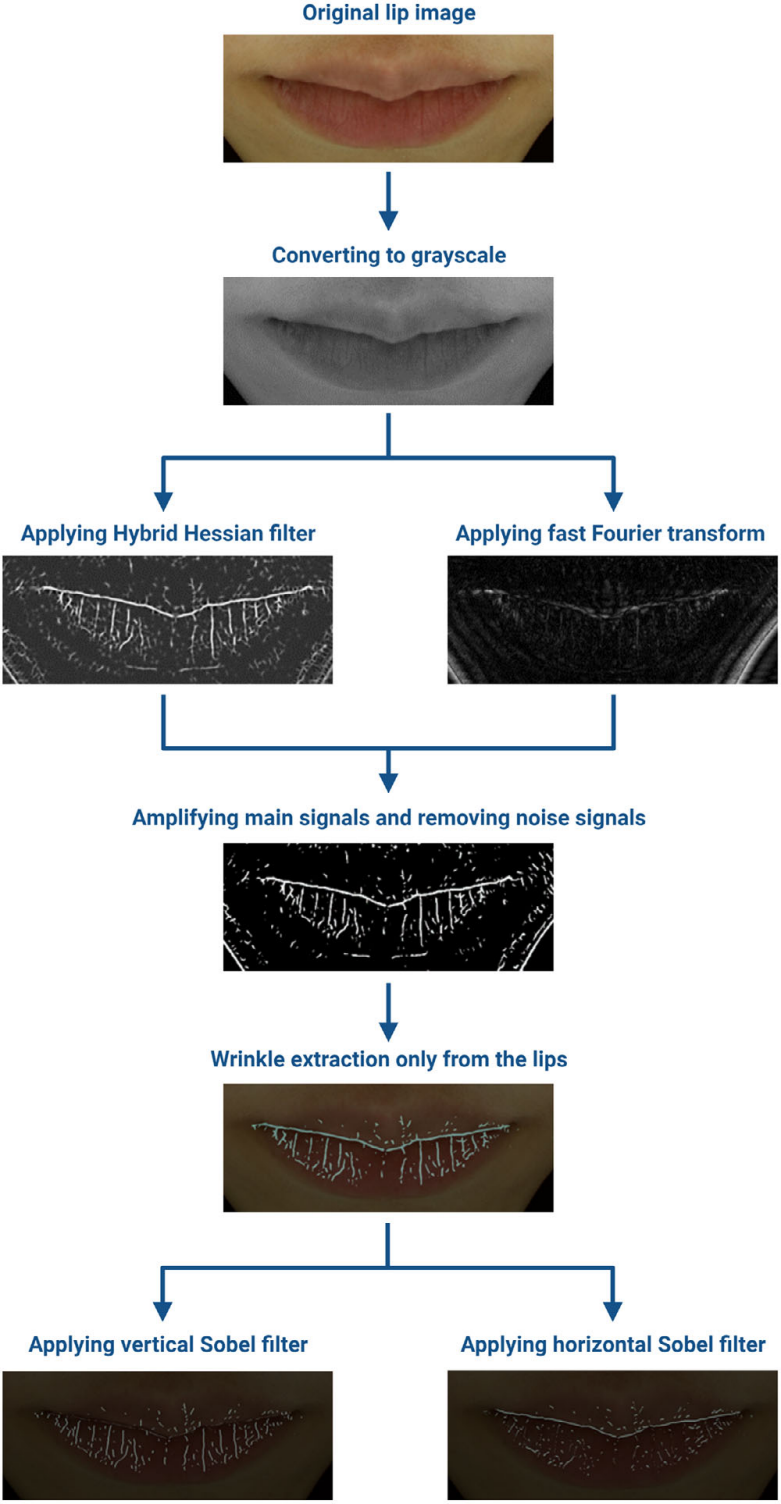
Overall flow chart of lip wrinkle extraction. The lip wrinkle indices were extracted based on a pattern recognition method, and representative images are included in the flow chart.

A total of 1000 Korean women were randomly selected, with 20 individuals per age ranging from 20 to 69 years old from our previous study.^20^ Frontal facial images were taken with a 24.2‐megapixel high‐resolution camera (Canon 200D DSLR, Canon, Tokyo, Japan) under a controlled visible light source installed in the Janus‐III measurement system (PIE, Suwon, South Korea). The institutional review board (IRB) at the LG H&H Research Center (Seoul, South Korea) approved this study. All participants were informed about this study and asked to sign an IRB‐approved written consent form. We obtained additional informed written consent from a participant included in Figures 1 and 2.

### 2.2 Lip image analysis

#### 2.2.1 Facial landmark detection and acquisition of lip image

We applied two image processing libraries, OpenCV^15^ and dlib,^14^ to detect landmarks on facial images obtained from 1000 participants. OpenCV was used for overall image processing tasks such as image load, save, crop, and transform, and dlib was utilized for facial landmark detection. As a result, a total of 68 facial landmarks were obtained from each facial image (Figure 1A). Among them, the landmarks corresponding to the contour of the lips are numbers 49–60, and we cropped the lip region using these points and renumbered for convenience of explanation (Figure 1B).

#### 2.2.2 Extraction of lip morphological indices using facial landmarks

Lip morphological indices were calculated using the detected lip region landmarks (Figure 1C). The upper vertical (UV) height indicates the longest vertical distance between upper and middle lip line (the average length of the vertical connection between landmarks 3 and 13 or 5 and 15), the lower vertical (LV) height indicates the longest vertical distance between middle and lower lip line (the average length of the vertical connection between landmarks 13 and 11 or 15 and 9), and lip width indicates the horizontal distance between left and right commissure (the length of the horizontal connection between landmarks 1 and 7) (Figure 1C). The lip ratio was calculated by the following equation:

$$\mathrm{Lip}\;\mathrm{ratio}\;=\;\frac{\mathrm{Lip}\;\mathrm{width}}{\mathrm{Upper}\;\mathrm{Vertical}\;\left(\mathrm{UV}\right)\;\mathrm{height}\;+\;\mathrm{Lower}\;\mathrm{Vertical}\;\left(\mathrm{LV}\right)\;\mathrm{height}\;}$$

#### 2.2.3 Extraction of lip wrinkle indices by pattern recognition method

The overall flow chart for lip wrinkle extraction is shown in Figure 2. First, the original images were converted into grayscale. Lip wrinkles were detected from the grayscale images using the ridge detection technique, hybrid Hessian filter.^18^ To enhance the quality of the detected signals, the main signals were extracted from the grayscale images using fast Fourier transform.^21^ Then images that applied hybrid Hessian filter were amplified by images that applied fast Fourier transform, to amplify main signals and remove noise signals. This process effectively enhanced the visibility and clarity of the detected lip wrinkles, providing a clearer representation. All parameters used for lip wrinkle detection were manually adjusted. The extracted wrinkles were factorized into horizontal and vertical wrinkles using a Sobel filter,^22^ and horizontal and vertical wrinkles were divided into upper and lower lip vermilion parts.

#### 2.2.4 Estimation of lip color as CIELAB color index

For lip color quantification, we used cropped images of the contour of the lips by using landmarks. The red values (the RGB values scaled to range 0–1) less than 0.3 in the lip image area were excluded to minimize the effect of the dark area of the images (the boundary between the upper and lower lips). Lip colors were calculated as the averages of red, green, and blue values and transformed to the CIELAB color index, L* (lightness), a* (redness), and b* (yellowness), which has been widely used for the quantification of objective color and is very close to human perception of color.^23^, ^24^, ^25^

### 2.3 Statistical analysis

Pearson correlation tests were conducted to assess the relationship between age and 11 lip characteristics, and also between lip morphological and wrinkle indices. All statistical analyses were performed with R software (version 3.6.3.), and the results were visualized using ggplot2 package.^26^

## 3 RESULTS

### 3.1 Evaluation of three lip aging features according to age

#### 3.1.1 Morphology

**FIGURE 3:**
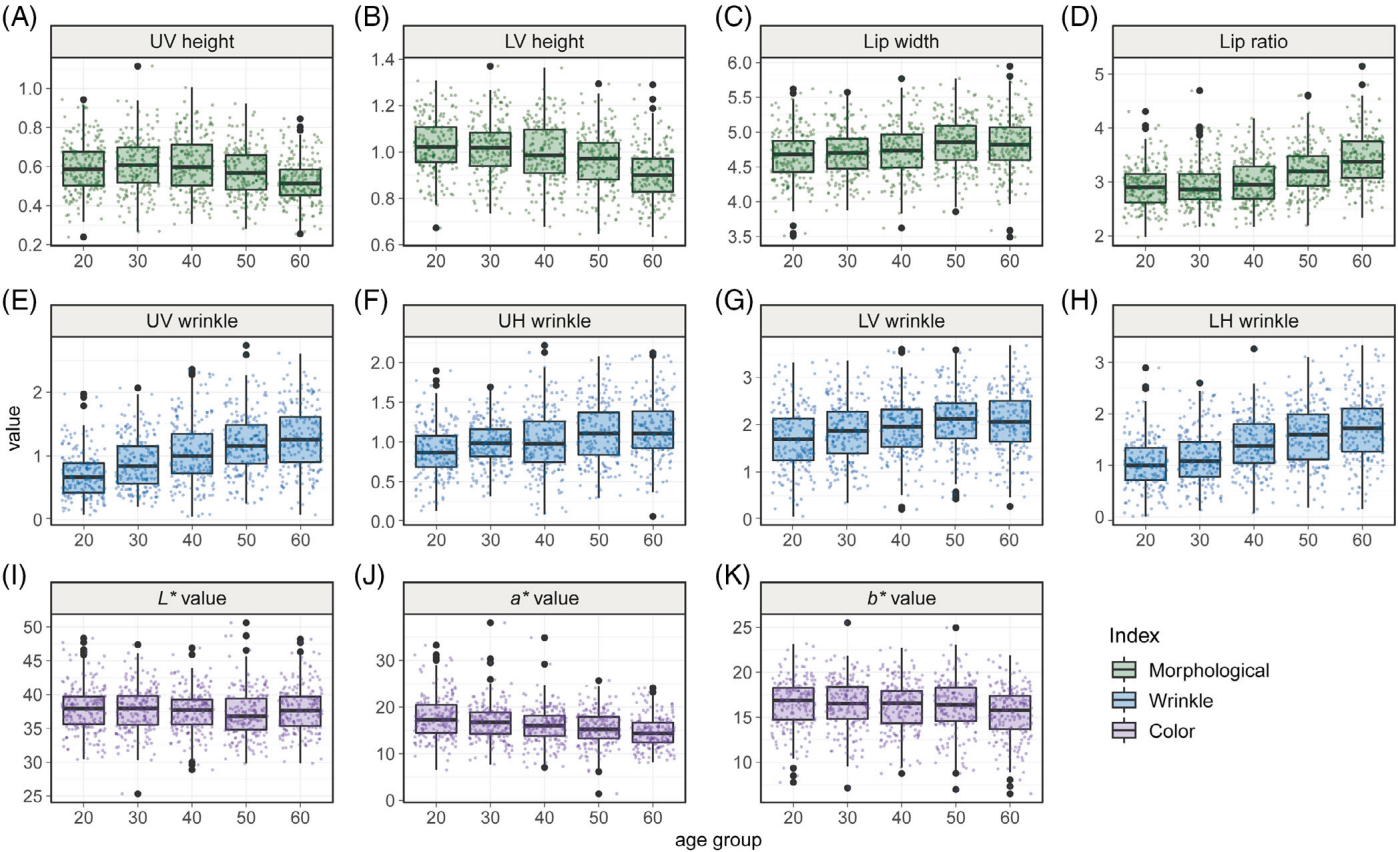
Lip characteristic indices according to age group. The lip morphological indices, upper vertical (UV) height, lower vertical (LV) height, lip width, and lip ratio are shown in (A)—(D). The lip wrinkle indices, upper vertical (UV) wrinkle, upper horizontal (UH) wrinkle, lower vertical (LV) wrinkle, and lower horizontal (LH) wrinkle are shown in (E)—(H). The lip color indices, CIE L*, a*, and b* values are shown in (I)—(K).

A total of 4 lip morphological indices including the upper vertical (UV) height, lower vertical (LV) height, lip width, and lip ratio according to age groups are shown in Figure 3A‐D and the average values are summarized in Table 1. Among the lip morphological indices, UV height and LV height indices were reduced (Figure 3A and 3B), while the lip width and lip ratio showed an increasing pattern with aging (Figure 3C and 3D). Especially, the UV height and LV height exhibited slight changes until the 40s, but thereafter decreased at a high rate. Moreover, the lip ratio remained relatively stable until the 40s, and then showed a high rate of increase.

**TABLE 1:** Comparison of the lip characteristic indices average by age group.

Index	Age group (mean)				
	20s	30s	40s	50s	60s
Lip morphological index					
UV height	0.60	0.61	0.61	0.57	0.52
LV height	1.03	1.02	1.00	0.96	0.91
Lip width	4.66	4.70	4.74	4.84	4.82
Lip ratio	2.91	2.94	3.00	3.21	3.42
Lip wrinkle index					
UV wrinkle	0.70	0.89	1.04	1.19	1.29
UH wrinkle	0.88	0.98	1.01	1.11	1.16
LV wrinkle	1.70	1.86	1.96	2.08	2.06
LH wrinkle	1.03	1.16	1.41	1.58	1.72
Lip color index					
L* value	37.9	37.8	37.5	37.2	37.8
a* value	17.8	16.8	16.1	15.5	14.6
b* value	16.5	16.5	16.3	16.2	15.5

Abbreviations: LH, lower horizontal; LV, lower vertical; UH, upper horizontal; UV, upper vertical.

**FIGURE 4:**
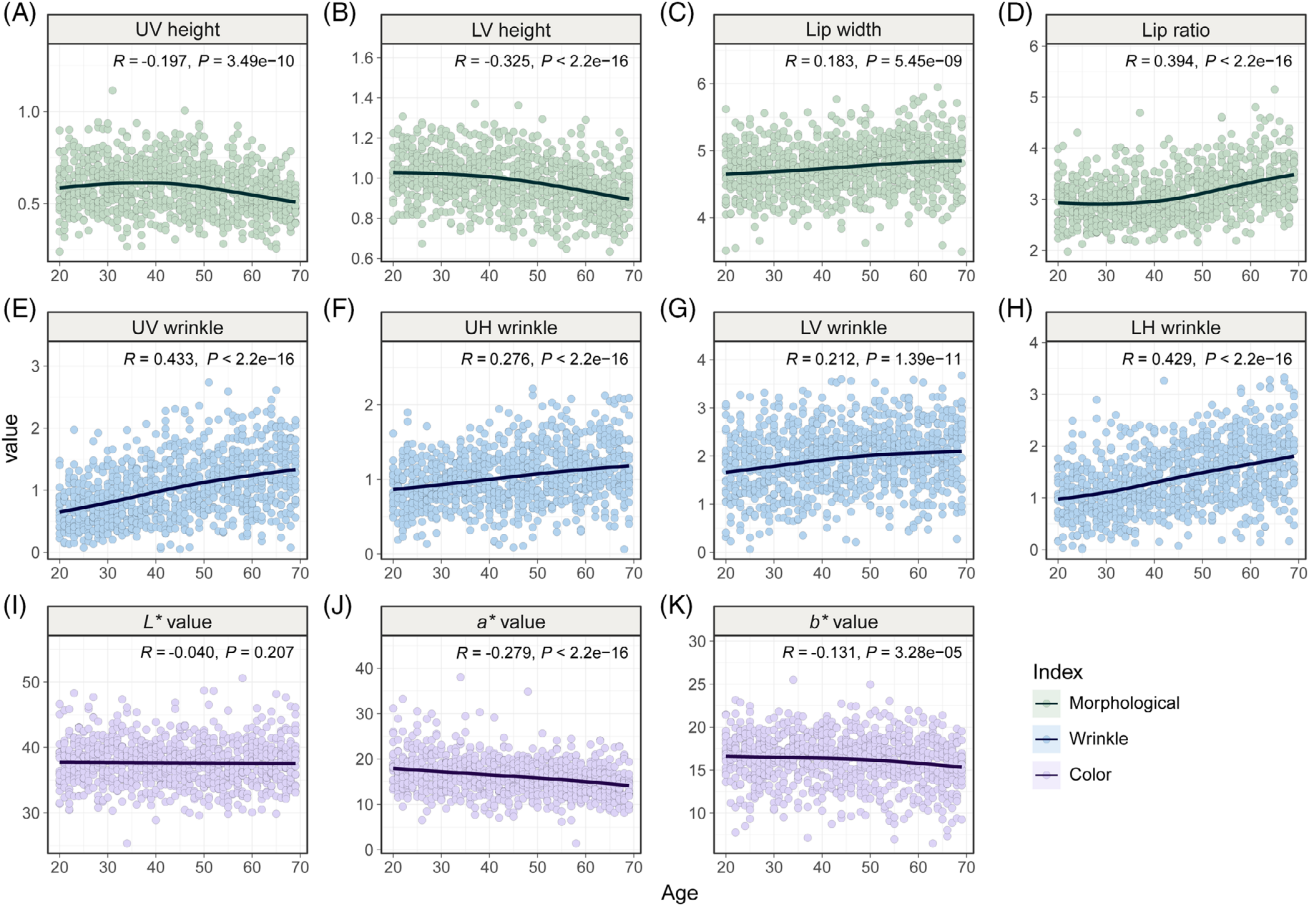
The scatter plots between lip characteristic indices and age. The lip morphological indices, upper vertical (UV) height, lower vertical (LV) height, lip width, and lip ratio according to age are shown in (A)—(D). The lip wrinkle indices, upper vertical (UV) wrinkle, upper horizontal (UH) wrinkle, lower vertical (LV) wrinkle, and lower horizontal (LH) wrinkle according to age are shown in (E)—(H). The lip color indices, CIE L*, a*, and b* values according to age are shown in (I)—(K). The given *R* values indicate Pearson correlation coefficient and *p* values represent the significance of the correlation test result.

A correlation analysis between lip morphological indices and age was also conducted (Figure 4A‐D). As a result, all lip morphological indices, UV height, LV height, lip width, and lip ratio were significantly correlated with age (*p* < 0.001), and the lip ratio showed the strongest correlation (correlation coefficient: 0.394).

#### 3.1.2 Wrinkles

The lip wrinkle indices were extracted by pattern recognition method, divided into upper and lower parts, and factorized into horizontal and vertical wrinkles. As a result, overall four lip wrinkle indices, upper vertical (UV) wrinkle, upper horizontal (UH) wrinkle, lower vertical (LV) wrinkle, and lower horizontal (LH) wrinkle were extracted, and lip wrinkles according to age group are shown in Figure 3E‐H and the average values are summarized in Table 1.

All lip wrinkle indices were positively correlated with age (*p* < 0.001). Interestingly, in the upper lip region, there was a tendency for UV wrinkle to increase with age more than UH wrinkle (correlation coefficient: 0.433 and 0.276, respectively), whereas in the lower lip region, LH wrinkle showed a higher correlation than LV wrinkle (correlation coefficient: 0.429 and 0.212, respectively) (Figure 4E‐H).

#### 3.1.3 Colors

Based on the CIELAB color space, CIE L*, a*, and b* were extracted from lip image and alterations according to age group are shown in Figure 3I‐K and the average values are summarized in Table 1.

Among the lip color indices, a* and b* values significantly correlated and decreased with aging (*p* < 0.05), while the L* value did not show significant variations with age. The most notable observation was the prominent reduction in the a* value, among lip color indices (correlation coefficient: −0.279) (Figure 4I‐K).

### 3.2 Correlation between lip wrinkle and morphological indices

**FIGURE 5:**
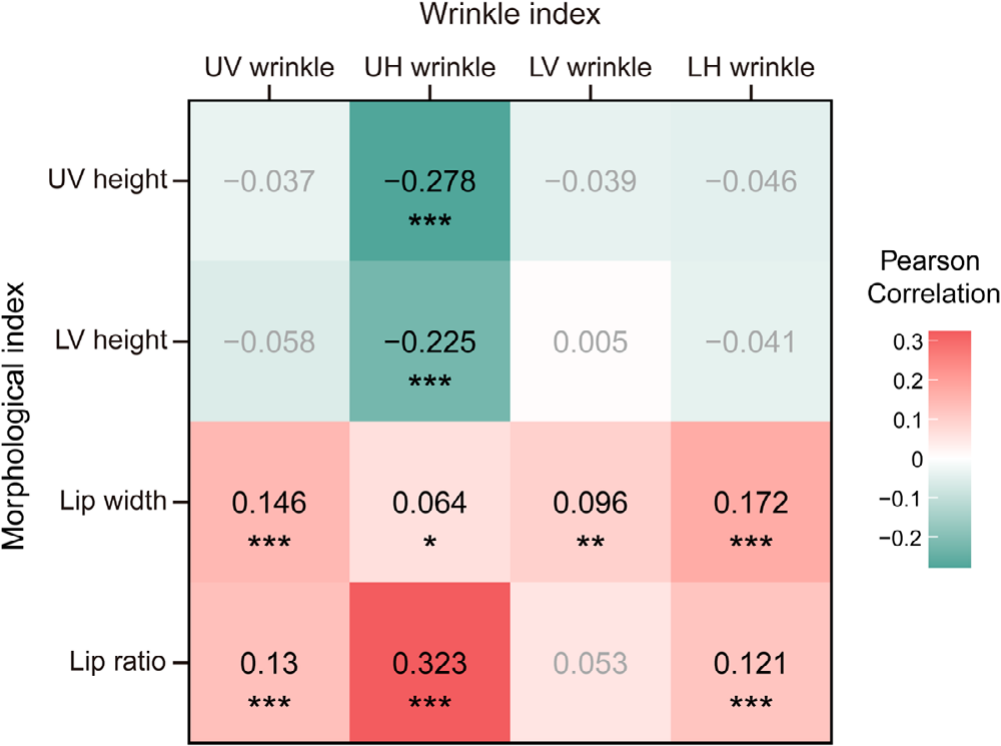
The correlation heatmap plot of lip morphological indices and wrinkle indices. Pearson correlations coefficient between lip morphological indices (upper vertical (UV) height, lower vertical (LV) height, lip width, and lip ratio) and lip wrinkle indices (upper vertical (UV) wrinkle, upper horizontal (UH) wrinkle, lower vertical (LV) wrinkle, and lower horizontal (LH) wrinkle) were calculated. Significant correlations are marked with stars, and non‐significant correlations are marked with gray (^*^ *p* < 0.05, ^**^ *p* < 0.01, and ^***^ *p* < 0.001). Red: positive correlations; green: negative correlations.

To investigate the underlying aging mechanism of human lip skin, we proceeded to analyze the correlation between lip wrinkle indices and lip morphological indices (Figure 5). All four lip wrinkle indices, UV wrinkle, UH wrinkle, LV wrinkle, and LH wrinkle, were significantly correlated with the lip width, respectively (*p* < 0.05), and among wrinkle indices, LH wrinkle showed the strongest correlation with the lip width. Moreover, three lip winkle indices, UV wrinkle, UH wrinkle, and LH wrinkle, were significantly correlated with the lip ratio, respectively (*p* < 0.001), and UH wrinkle had the highest correlation with the lip ratio. Especially, two morphological indices of lip thickness, UV height and LV height, showed a significant negative correlation only with UH wrinkle, respectively (*p* < 0.001), and UV height had a slightly stronger correlation coefficient than LV height (correlation coefficient: −0.278 and −0.225, respectively).

## 4 DISCUSSION AND CONCLUSION

The lips constitute a significant facial feature that greatly influences an individual's facial aesthetics. Consequently, understanding the aging characteristics specific to the lips becomes a necessary endeavor. However, to our knowledge, comprehensive studies on lip aging using considerable lip images are currently lacking. Therefore, this study aimed to extract lip characteristics indices from facial images of 1000 Korean women and investigate the patterns of lip aging based on these indices.

Firstly, age‐related morphological changes in the lips were observed across three factors: lip height, lip length, and lip ratio extracted based on the facial landmark detection method. The decrease in lip thickness, or in other words, the height of the lip, with aging, is a well‐established result in previous studies.^6^, ^27^ Otherwise, Kim et al. reported that the height of the lip showed no significant differences according to age group.^13^ Our study confirmed the trend of lip thinning (decreasing lip height) with aging, and further revealed that the decrease in LV height exhibited a more distinct tendency compared to UV height (correlation coefficient: −0.325 and −0.197, respectively). Since the decrease in lower lip thickness is a prominent feature of aging,^28^ focusing on the lower lip could be a considerable approach in lip aging care strategy. Although a previous study reported no changes in lip width with age,^29^ the increase in lip length with aging has been elucidated through the majority of previous studies,^4^, ^5^, ^13^ and our large‐scale image study also confirms this trend. These morphological changes of the lips are closely related to muscle alterations associated with aging. The orbicularis oris muscle, which is attached to the dermal layers of both the upper and lower lips, serves as a point of attachment for several other facial muscles in the perioral region. According to aging, this muscle has been known to undergo thinning and morphological transformations, across sections of the upper lip, orbicularis oris muscle shifting from a J‐shape to an L‐shape, accompanied by an increase in angle.^5^ Among the lip morphological indices, a lip ratio was formulated by incorporating both lip height and lip width indices. Notably, when considering the correlation of each index with age, the lip ratio exhibited the highest correlation coefficient, which suggests that the lip ratio can be used as an indicator of lip aging features such as lip tension, tightening, and sagging.

Previous studies on wrinkles close to lip tissue have predominantly focused on perioral wrinkles rather than vermillion zone wrinkles.^7^, ^30^ In this study, we aimed to ascertain lip vermilion wrinkles by extracting wrinkles from images by pattern recognition method. Furthermore, we subdivided the lips into upper and lower regions, allowing us to intricately examine the detailed patterns of lip aging by categorizing the wrinkles within each region as horizontal and vertical wrinkles. While a small‐scale Korean study reported that the older group had decreased lip wrinkles compared to younger group,^13^ our results revealed that lip wrinkle indices were positively correlated with age. The differences in the results between the previous study and this study are presumed to be due to variations in wrinkle measurement equipment and wrinkle extraction algorithms. Considering the limited research on lip vermilion zone wrinkles, it would be necessary to conduct further studies with diverse cohorts. To our knowledge, our investigation is the first to segment and analyze lip wrinkles from a large‐scale image dataset. Our findings revealed that in the upper lip, vertical wrinkles showed a stronger correlation with age than horizontal wrinkles. Conversely, in the lower lip, horizontal wrinkles exhibited a stronger correlation with age than vertical wrinkles. As it suggests that the cause of wrinkles formation can be varied depending on the upper and lower regions of the lips, further related functional studies are needed.

Lip color indices, as indicated by the a* and b* values, showed age‐associated decrease. The observed change in redness (CIE a* value) can be partially supported by a histological research, which reported that decrease in the number of vessels in the lip vermilion with age.^31^ While previous small‐scale studies have reported no significant correlation between the b* value and aging,^11^, ^13^ our large‐scale study has revealed a negative correlation between lip yellowness and age. Although previous study showed that skin yellowness is related to a healthy and attractive appearance (CIE b* value),^32^ there has been no exploration of such studies on lip skin. Further functional studies of decreasing yellowness with aging in terms of molecular biology and genetics are also required to elucidate their contributions to lip aging. While several studies reported a decrease in the L* value of the lower lip with age,^11^, ^33^ and some studies showed age‐related differences only in the upper lip,^13^ we did not observe a correlation between the L* value and age in our large‐scale dataset. The lack of correlation can be attributed to the low melanin content in lip vermilion.^34^, ^35^ Whereas research on lip brightness and melanin has been limited, a recent study has reported a correlation between the brightness of the lower lip and melanin.^35^ Therefore, additional functional studies are required. This will enhance our understanding of the relationship between lip brightness and melanin content. We conducted additional analyses on lip color indices separately for the upper and lower lips. Although the a* and b* values of the lower lip exhibited stronger correlations with aging and were statistically significant (with correlation coefficients of −0.157 and −0.306 for the a* value, and −0.066 and −0.133 for the b* value, respectively), the overall trends in lip color changes with age remained consistent. The discrepancies observed in aging and lip color trends in our study can be attributed to the utilization of a different lip color measurement method compared to previous studies. Most of the previous studies utilized a spectrophotometer or chromameter to measure only small areas of the lips (0.20–0.50 cm^2^).^36^, ^37^ To address the limitation of measuring only a small area, we employed landmark‐based techniques to extract colors from the entire lip images. This allows us to represent the overall lip color more accurately. Given the various factors such as melanin, blood vessels in the lip, and others that affect the color of the lips, further researches are necessary to explore the correlation between lip color and aging in detail.

The correlation analysis between the indices provided important aspects of lip aging. All the lip wrinkle indices showed positive correlations with lip width and three of them were also correlated with lip ratio. Moreover, UH wrinkles were negatively correlated with the indices representing lip thickness, UV height, and LV height. Such results imply that a potential interaction may exist between age‐associated lip thinning and wrinkle formation. Considering the reported association between lip thinning and oris muscle thinning,^38^ the mechanism of wrinkle formation in lip tissue may be explained by muscular atrophy. Further studies about muscular aspects will contribute to a comprehensive understanding of the process of lip aging.

In this study, we explored the visual changes of lip morphologies, wrinkles, and color indices according to aging in a large‐scale Korean population. The observed lip morphological changes, such as lip height shortening and lip width increasing, were consistent with previous reports. Among the morphological indices, the lip ratio demonstrated the strongest correlation with aging and can be utilized as a reliable indicator of lip aging. All the lip vermilion wrinkles increased, while redness and yellowness decreased with aging. Additional correlation analysis suggested meaningful insight about the possible associations between morphological changes and wrinkle formation of lips. Our study could contribute to a deeper understanding of lip aging and the development of new technologies and treatments in the fields of cosmetics, dermatology, and plastic surgery.

## CONFLICT OF INTEREST STATEMENT

The authors state no conflict of interest.

## ETHICS STATEMENT

The institutional review board (IRB) at the LG H&H (Seoul, South Korea) research center approved this study. All research was conducted in accordance with relevant guidelines and regulations. All participants were informed about this study and were asked to sign an IRB‐approved written consent form (2020‐GR‐013). We obtained an additional informed written consent form from a participant included in the figures.

## Data Availability

The data that support the findings of this study are available on request from the corresponding author. The data are not publicly available due to privacy or ethical restrictions.
